# The Outcomes of Patients with Omicron Variant Infection who Undergo Elective Surgery: A Propensity-score-matched Case-control Study

**DOI:** 10.7150/ijms.90695

**Published:** 2024-03-17

**Authors:** Xiaojuan Xiong, Rui Li, Hong Yan, Qingxiang Mao

**Affiliations:** Department of Anesthesiology, Army Medical Center of PLA, Daping Hospital, Army Medical University, 10 ChangjiangZhilu, Yuzhong District, Chongqing 400042, China.

**Keywords:** Omicron variant, postoperative complications, timing of surgery

## Abstract

**Aim:** To investigate whether it is safe for patients with Omicron variant infection to undergo surgery during perioperative period.

**Methods:** A total of 3,661 surgical patients were enrolled: 3,081 who were not infected with the Omicron variant and 580 who were infected with the Omicron variant. We conducted propensity score matching (PSM) with a ratio of 1:4 and a caliper value of 0.1 to match the infected and uninfected groups based on 13 variables. After PSM, we further divided the Infected group (560 cases) by the number of days between the preoperative Omicron variant infection and surgery: 0-7, 8-14, 15-30, and >30 days. Multivariate logistic regression analysis was subsequently conducted on the categorical variables and continuous variables with a *P* value below 0.05, thereby comparing the infected group (0-7, 8-14, 15-30, >30 days) and the uninfected group for perioperative complications.

**Results:** Multivariate logistic regression analysis revealed that, compared to the uninfected group, among the four subgroups of the infected patients (0-7, 8-14, 15-30, >30 days), only renal insufficiency in the 8-14 days subgroup (OR: 0.09, 95%CI 0.01-0.74, *P* = 0.025) and anemia in the > 30 days subgroup (OR 0.6, 95%CI 0.4-0.9, *P <* 0.017) showed significant difference. However, there was no statistically significant difference in the incidence rate of blood transfusion, postoperative intensive care unit transfer, lung infection/pneumonia, pleural effusion, atelectasis, respiratory failure, sepsis, postoperative deep vein thrombosis, hypoalbuminemia, urinary tract infections, and medical expenses.

**Conclusion:** Omicron infection does not significantly increase the risk of perioperative major complications. The Omicron infection may not be a sufficient risk factor to postpone elective surgery.

## Introduction

COVID-19 is a respiratory illness characterized by its sudden onset, caused by a specific coronavirus with a genetic material composed of single-stranded RNA and distinctive spike proteins on its surface 1. The Omicron variant (B.1.1.529) is a worrisome, newly emerged form of the COVID-19 virus. It was initially detected in South Africa on November 26, 2021. Subsequently, this variant caused a significant surge in COVID-19 cases in Europe and the United States 2. To November 8, 2023, there have been more than 771 million confirmed cases of COVID-19 worldwide, resulting in approximately 6.98 million deaths 3. On January 8, 2022, Tianjin confirmed the first indigenous case of Omicron variant infection. The variant then spread across China 4. In December 2022, there was a rapid surge in the number of Omicron variant infections 5 as the country loosened its control over COVID-19. In Chongqing, the dominant variant during that period was Omicron-BA.5.2 variant 6.

Maslo et al. observed that compared with patients infected with previous variants, those infected with the Omicron variant are generally younger, have fewer comorbidities, and experience lower severity and mortality rates 7. Furthermore, asymptomatic infections and mild symptoms are more common among those infected with this variant 7. Early research by Madhi SA et al. suggested that its pathogenicity was greatly reduced, and the morbidity and mortality were greatly reduced during the widespread transmission of the Omicron variant 8. Severe acute respiratory syndrome coronavirus-2 (SARS-CoV-2) is a rapidly evolving RNA virus that has recently mutated to form the Omicron variant. Compared to the Delta variant, the Omicron variant exhibits a significantly higher replication rate and notably greater infectivity 9. According to the Association of Anesthetists in England, no evidence has been found to suggest how infection with the Omicron variant after receiving the SARS-CoV-2 vaccine might impact perioperative outcomes. Therefore, the advice to minimize elective surgery within seven weeks of contracting SARS-CoV-2 infection is still valid 10. Sridhar et al. reported that individuals who have recuperated from asymptomatic or mild COVID-19 infections can safely undergo elective general surgical procedures after a waiting period of at least two weeks 11. While a study conducted by Lieberman Nd et al. suggests that elective surgery can safely take place with 10 days delay from either the first day of symptom onset or the day of the first positive SARS-CoV-2 test result 12. Dobbs TD et al. highlighted that asymptomatic patients who have been vaccinated can safely proceed with elective surgery within 5 to 10 days after being diagnosed 13.

Most of currently available studies suggest that patients infected with the Omicron variant ideally undergo surgery with a delay of 5 days to 7 weeks 10,11,12,13. In the present study, we categorized our patients into four groups by time of surgery relative to their first infection (0-7, 8-14, 15-30, and >30 days), and compared the rate of perioperative complications, aiming to determine the best timing of surgery in patients with Omicron variant infection.

## Materials and methods

### Ethics approval

As this is a retrospective study, the Ethics Committee waived the requirements of obtaining the Informed Consent Forms from the patients. On May 4, 2023, the Army Medical Center of the PLA approved this study, with ratification number 2023 177.

### Registration

The study registered in the WHO International Clinical Trial Registration (ChiCTR2300071913).

### Inclusion and exclusion criteria

Inclusion criteria: All patients who had elective surgery at our hospital between October 2022 and January 2023.

Exclusion criteria: patients with no medical records pertaining to the Omicron variant between December 2022 and January 2023; patients under 18 years of age.

### Research method

**Figure 1:**
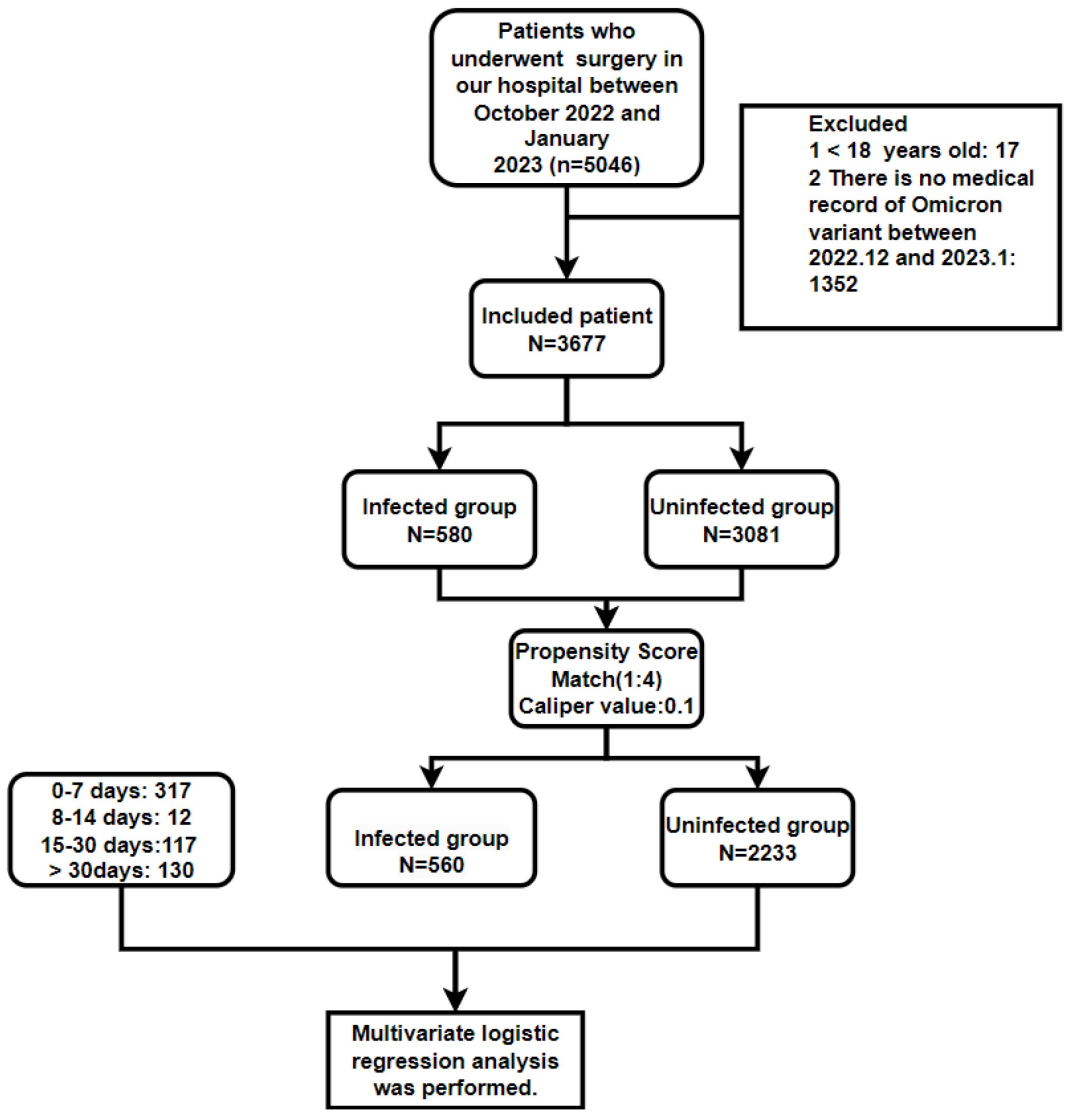
Research scheme of propensity score matching analysis of uninfected and infected with Omicron variant in patients undergoing surgery.

In total, 3,677 patients underwent surgery, comprising 3,081 uninfected patients (from October 2022 to January 2023) and 580 infected patients (from December 2022 to January 2023). Upon hospital admission, each patient underwent an rt-Polymerase chain reaction (PCR) test utilizing a nasopharyngeal swab specimen. Subsequently, patients were categorized into infected (Control group) and non-infected (Experimental group) groups based on these test results. The infected group and the non-infected group underwent propensity score matching (PSM) based on various factors, including age classification (≥ 60 years, < 60 years), gender (female, male), fractures, malignant tumors, hypertension (HBP), coronary heart disease (CHD), diabetes (DB), cerebral infarction, chronic obstructive pulmonary disease (COPD), preoperative deep vein thrombosis (DVT), surgical type (emergency surgery or elective surgery), methods of anesthesia (general anesthesia, spinal anesthesia, nerve block, local anesthesia), and American Society of Anesthesiologists (ASA) classification (I, II, III, IV, V) (**Figure 1**).

After PSM, the Infected group was further subdivided into four subgroups based on the time interval between Omicron variant infection and surgery: 0-7, 8-14, 15-30, and >30 days. A comparative analysis was subsequently performed between the Infected group and the Uninfected group in the following aspects: blood transfusion, postoperative patient transfer (regular ward or intensive care unit (ICU)), postoperative diagnosis (lung infection/pneumonia, pleural effusion, atelectasis, respiratory failure, renal insufficiency, sepsis, postoperative DVT, hypoalbuminemia, anemia, urinary tract infection), and medical expenses.

### Data collection

We primarily acquired patients' clinical data by querying our hospital's clinical electronic medical record system and the relevant anesthesia-surgical systems. The collected information included: age, gender; preoperative medical records: HBP, diabetes, CHD, COPD, cerebral infarction, bone fracture, malignant tumor, preoperative DVT; during the surgery: surgery type (emergency or elective), method of anesthesia (general, spinal, or local anesthesia; nerve block), ASA classification, utilization of blood transfusion, postoperative patient transfer: regular ward or ICU; postoperative diagnosis: lung infection/pneumonia, pleural effusion, atelectasis, respiratory failure, renal insufficiency, sepsis, postoperative DVT, hypoalbuminemia, anemia, urinary tract infection, postoperative DVT, and medical expense. For the Infected group, the time interval between first Omicron variant infection and elective surgery was also collected. The diagnosis criteria for Omicron variant infection by our laboratory are as follows: (i) Before surgery, a positive rapid antigen test was conducted or a positive RT-PCR nasopharyngeal swab was performed; (ii) The preoperative chest computed tomography (CT) scan revealed findings consistent with pneumonitis associated with SARS-CoV-2 infection; (iii) A preoperative test indicating the presence of positive immunoglobulin G (IgG) or immunoglobulin M (IgM) antibodies 14. According to the WHO criteria (and the reference values adopted by our laboratory), hemoglobin levels below 130 g/L for males or below 120 g/L for females are classified as anemia 15.

### Statistical analyses

Statistical analysis was conducted by using SPSS26.0 (IBM Corp, Armonk, NY, USA). Patients after matching were categorized into the Infected group and the Uninfected group. PSM was subsequently employed for both groups with a ratio of 1:4 and a caliper value of 0.1. Categorical variables were compared using the chi-square test, while continuous variables were compared using the ANOVA test. Normally distributed enumeration data were represented by the mean plus or minus standard deviation (x ± s), and skewed enumeration data were represented by median (quaternary) [M (Q1, Q3)]. Multivariate logistic regression analysis was subsequently conducted on the categorical variables and continuous variables with a P value below 0.05, thereby comparing the infected group (0-7, 8-14, 15-30, >30 days) and the uninfected group for perioperative complications. *P* < 0.05 was considered statistically significant.

## Results

### Basic patient information

A total of 3,661 patients were enrolled (**Figure 1**). Before PSM, there were 2,153 males and 1,508 females, with a mean age of 49.5 years (in the Infected group) and 49.2 years (in the Uninfected group). There were 282 patients with bone fracture, and 979 patients with malignant tumor. The most common preoperative comorbidities in the patients included HBP (580 cases), DB (372 cases), and CHD (107 cases) (**Table 1**). Ages in each group after PSM are shown in **Table 2**.

### Results of PSM analysis

PSM was applied to the Infected and Uninfected groups based on 13 variables: gender, age group, bone fracture, malignant tumor, HBP, DB, CHD, COPD, cerebral infarction, preoperative DVT, emergency or elective surgery, anesthesia method, and ASA classification. Results revealed that all variables had a *P* value > 0.05 and none had a standard deviation (**|**d**|**) greater than 0.25, suggesting that all the variables were balanced after the matching (**Table 3**). After PSM, the Infected group had 560 cases and the Uninfected group had 2,231, enabling a well-balanced comparison between the two groups.

### Results of multivariate logistic regression analysis

**Figure 2:**
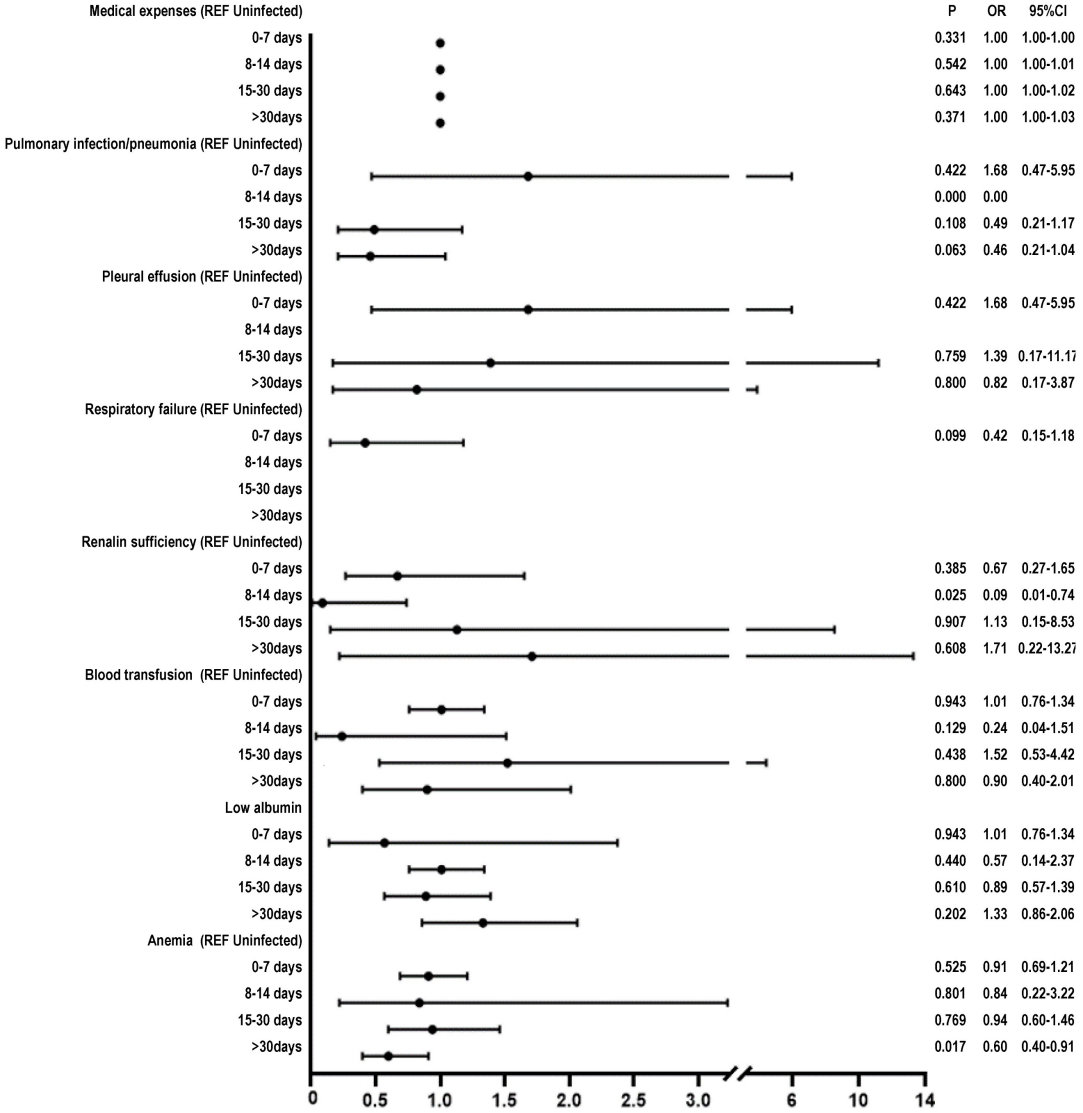
Multivariate logistic regression was performed to compare postoperative outcomes between SARS-CoV-2 negative patients and preoperatively positive SARS-CoV-2 patients stratified by timing of positivity before surgery with complications, as compared to SARS-CoV-2 negative patients after PSM.

After applying ANOVA test to the data following PSM, we observed a significant difference between the groups on the variable “medical expenses” (*P* < 0.05). We further performed Chi-square test and found that the following variables showed between-group differences (*P* < 0.05): pulmonary infection/pneumonia, low albumin, anemia, respiratory failure, renal insufficiency, and blood transfusion. The multivariable logistic regression analysis revealed the following results: renal insufficiency in the 8-14 days subgroup (OR: 0.09, 95% CI 0.01-0.74, *P* = 0.025) and in the > 30 days subgroup (OR 0.6, 95%CI 0.4-0.9, *P* < 0.017) (**Figure 2**).

Our study revealed no significant difference in the rate of perioperative complications (blood transfusion, postoperative ICU transfer, lung infection/pneumonia, pleural effusion, atelectasis, respiratory failure, sepsis, postoperative DVT, hypoalbuminemia, urinary tract infection, and medical expenses) in surgical patients with Omicron variant infection (0-7, 8-14, 15-30, > 30 days), as compared to uninfected patients. Although significant difference was observed in renal insufficiency for 8-14 days subgroup and anemia for > 30 days subgroup, it makes little difference.

## Discussion

The global pandemic has exerted enormous pressure on healthcare systems worldwide, resulting in a sharp reduction in surgical activity. Within the first 12 weeks of pandemic outbreak 3, approximately 28 million surgeries were canceled, leading to millions of patients who were still in anticipation of their scheduled procedures 16. Barie et al. emphasized that following the COVID-19 pandemic, there is an anticipated substantial increase in surgical procedures, potentially straining healthcare workers. Prolonged and distressing delays for particular patients may occur, ultimately resulting in frustration among surgical teams 17. Given the persistent mutations of COVID-19 variants, alterations in transmissibility, virulence, and mortality rates are ongoing. Consequently, the scheduling of postponed surgeries should be adjusted accordingly 18. El-Boghdadly et al. reported that perioperative risk returned to baseline at 7 weeks after SARS-CoV-2, prompting their recommendation for a 7-week delay post-infection before surgery 10. However, it is crucial to recognize that none of the variants they investigated were the Omicron variant. In another study, Baiocchi G et al. found that for patients who had recovered from asymptomatic and mild COVID-19 infections, undergoing oncological surgeries after a waiting period of at least 2 weeks, with a median time of approximately 25 days (ranging from 12 to 84 days) following their COVID-19 diagnosis, did not result in a significantly higher risk of postoperative complications when compared to patients without COVID-19 17. However, it's worth mentioning that their study had a relatively limited sample size, consisting of just 49 cases. Codner et al. found that the timing of preoperative SARS-CoV-2 positivity relates to severe complications during the perioperative period. Their study involved 262 SARS-CoV-2 positive and 1,840 negative patients.

In the 0-15 day timeframe, SARS-CoV-2 positive patients were 1.88 times more likely to experience severe complications compared to SARS-CoV-2 negative surgical patients; within 15-30 days, it reduced to 0.43-fold, and within 31-50 days, it was 0.98-fold when compared to their SARS-CoV-2 negative counterparts 19. In certain circumstances, they have found that postponing surgery by 14 days after testing positive for SARS-CoV-2 may be advisable 20. But the sample size in their study is relatively small, potentially leading to biased results. Glasbey et al. reported that the time gap between asymptomatic SARS-CoV-2 infection and elective surgery could potentially be reduced to 5 days, but there is no supporting evidence from surgery-receiving patients 21.

In contrast to these studies, our research is the first to reveal that patients infected with the Omicron variant (at different intervals post-infection, including 0-7, 8-14, 15-30, and >30 days) undergoing surgical treatment did not exhibit significant difference in perioperative complication rate (including transfusions, postoperative ICU admissions, lung infections/pneumonia, pleural effusion, lung collapse, respiratory failure, sepsis, postoperative deep vein thrombosis, hypoalbuminemia, urinary tract infections, and medical expenses), as compared to uninfected patients. We also observed significant difference in renal insufficiency in the 8-14 days subgroup (OR 0.09, 95% CI 0.01-0.74, *P* = 0.025) and anemia in the >30 days subgroup (OR 0.6, 95% CI 0.4-0.9, *P <* 0.017). In COVID-19 patients, the immune response to the viral infection can lead to elevated levels of serum cytokines, including Interleukin-6 and Tumor necrosis factor. These cytokines exhibit direct nephrotoxic effects 22,23. Teng et al. observed that there was a mild decline in renal function after Omicron variant infection and returned to normal within six months. And most patient infected with Omicron variant exhibited a mild inflammation response 24. Lechner-Scott et al. found that post-COVID-19 patients suffered persistent inflammation response 25, which may affect RBC production and RBC lifespan, and finally lead to the development of inflammatory anemia 26. The mild inflammation observed after Omicron infection 24 results in a relatively minor effect on renal insufficiency and anemia. Therefore, it appears that Omicron variant infected patients do not need to postpone their surgery, whether it is elective or emergency. The reasons will be further explored below from the perspective of the Omicron variant virus and vaccination against COVID-19.

### Omicron variant virus

Early research by South African scientists indicated that during the widespread transmission of the Omicron variant, its pathogenicity has significantly diminished, resulting in a substantial decrease in the occurrence of severe cases and mortality rate 8. SARS-CoV-2, similar to other RNA viruses, can undergo mutations and genetic evolution over time as it adjusts to new human hosts, potentially leading to mutant variants exhibiting distinct characteristics compared to their original strains 27. SARS-CoV-2 exhibits a relatively high mutation rate, and by 2022, several variant strains 28. The Omicron strain has demonstrated higher transmissibility, evading immune system defenses, and exhibiting limited susceptibility to the COVID-19 vaccine 29. Nevertheless, recent studies have reported that compared to other SARS-CoV-2 pathogen infections, Omicron variant infections are closely associated with a higher incidence of asymptomatic carriage, milder symptoms, lower hospitalization rate, and mortality 30. Emerging data indicate that the Omicron variant tends to result in a higher number of asymptomatic cases, less severe symptoms, and lower rates of hospitalization and mortality when compared to previous variants 31. In the analysis conducted by Desai et al., researchers have made a notable observation: the mortality rate among cancer patients diagnosed with COVID-19 during the Omicron phase is considerably lower than that of patients diagnosed during the early stages of the pandemic, with the acute mortality rate dropping from over 30% to below 10% 32.

### Association with past COVID-19 vaccination

Cortellini et al. reported that previous SARS-CoV-2 immunization is an effective measure in safeguarding patients from COVID-19 sequelae, minimizing treatment disruptions, and reducing mortality rate 33. Christensen et al. found that within their healthcare system, Omicron patients were notably younger, exhibited significantly higher vaccination rates, and had a significantly lower likelihood of hospitalization when compared to patients infected with the Alpha or Delta variants 31. Furthermore, the Omicron variant group needed less intensive respiratory support and required shorter hospitalizations, aligning with an overall reduction in disease severity 31. Moreover, it has been widely demonstrated that vaccinated surgical patients experience significantly reduced COVID-19 mortality compared to the general vaccinated population 34.

Large-scale vaccination campaigns have been implemented worldwide over the past two years to prevent and control the transmission of the Omicron variant. Specifically, the most widely administered SARS-CoV-2 vaccine is the inactivated one in mainland China 35. Although domestic vaccines may not provide complete protection against Omicron variants, they do offer significant safeguards for adult patients 36. Wang J et al. found that vaccinated adult patients had notably lower levels of Interleukin-6 and C-reactive protein compared to unvaccinated patients, revealing that the domestic vaccines provide significant protection against inflammation reduction in adult patients during the recovery phase 36. In our study, over 90% of the patients had been vaccinated, which likely contributed to a notable reduction in the rate of perioperative complications. The Omicron variant has notably increased its transmissibility and possesses the capability to evade immunity acquired through previous infections, vaccination, or a combination of both 37. Pilz et al. noted that compared to 2020, the severity of SARS-CoV-2 infections has noticeably diminished, with a significant reduction in associated complications. These trends may be attributed to the reduced virulence of prevalent variant strains, vaccination programs, and the natural immunity developed from previous infections 38. Vaccination against COVID-19 stands as the most effective measure to reduce the severity of infection and minimize perioperative complications. Therefore, it is strongly encouraged to promote vaccination preoperatively 36. Thus, Omicron variants exhibit high transmissibility but lower pathogenicity compared to other SARS-CoV-2 variants. Additionally, vaccination and previous infections have significantly enhanced population immunity, resulting in a decrease in severe cases of COVID-19. Consequently, SARS-CoV-2 is anticipated to circulate in a manner similar to seasonal coronaviruses 39.

In conclusion, the Omicron variant has reduced pathogenicity as it constantly evolves, leading to gradually attenuated inflammation within the body. In addition, as patients are generally vaccinated against SARS-CoV-2 and tend to be younger, the patients showed no significant difference in the rate of perioperative complications undergoing surgery at 0-7, 7-14, 14-30, and >30 days after infection, as compared to uninfected surgical patients. It is relatively safe for Omicron variant patients to undergo surgery, as the rate of perioperative complications showed no significant difference, thus maybe there is no need to postpone surgery.

We conducted this retrospective analysis primarily by collecting medical history and laboratory tests of patients who were uninfected or infected with the Omicron variant and underwent surgery. Nevertheless, there are certain limitations to this study. Firstly, it is a retrospective study and thus some data may be incomplete, including missing information in the medical records of some patients. In addition, because it is a single-center, retrospective, case-control study, there may be potential unaccounted confounding factors, necessitating further research with larger sample sizes.

## Conclusion

We found no significant differences in the incidence of perioperative complications among patients with Omicron variant infection undergoing surgery at 0-7, 8-14, 15-30, and >30 days after infection compared to the uninfected patients.

## Figures and Tables

**Table 1:** Distribution characteristics of covariates in surgical patients before and after PSM in the Infected group and Uninfected group

	Before matching (3661)						After matching (2824)					
Covariates	Infected group (580)		Uninfected group (3081)		*X^2^*	*P*	Infected Group (560)		Uninfected group (2233)		*X^2^*	*P*
Gender											0.34	0.297
Male	425	73.28%	2231	72.41%	0.70	0.22	327	58.39%	1334	59.74%		
Female	155	26.72%	850	27.59%			233	41.61%	899	40.26%		
Age group (year)					0.18	0.36					1.59	0.114
≤60	464	80.00%	2231	72.41%			412	73.57%	1700	76.13%		
>60	86	14.83%	850	27.59%			148	26.43%	533	23.87%		
Fracture					6.77	0.01					1.83	0.104
Yes	60	10.34%	222	7.21%			57	10.18%	187	8.37%		
No	520	89.66%	2859	92.79%			503	89.82%	2046	91.63%		
Malignant tumor					0.25	0.325					0.34	0.219
Yes	160	27.59%	819	26.58%			158	28.21%	608	27.23%		
No	420	72.41%	2262	73.42%			402	71.79%	1625	72.77%		
HBP					0.09	0.402					0.98	0.178
Yes	86	14.83%	442	14.35%			84	15.00%	299	13.39%		
No	494	85.17%	2639	85.65%			476	85.00%	1934	86.61%		
DB					1.84	0.101					0.13	0.381
Yes	68	11.72%	304	9.87%			65	11.61%	247	11.06%		
No	512	88.28%	2777	90.13%			495	88.39%	1986	88.94%		
CHD					0.30	0.33					0.25	0.353
Yes	19	3.28%	88	2.86%			18	3.21%	63	2.82%		
No	561	96.72%	2993	97.14%			524	93.57%	2170	97.18%		
COPD					0.06	0.462					0.2	0.385
Yes	10	1.72%	49	1.59%			10	1.79%	34	1.52%		
No	570	98.28%	3032	98.41%			550	98.21%	2199	98.48%		
Preoperative DVT					0.18	0.396					0.65	0.27
Yes	8	1.38%	36	1.17%			8	1.43%	23	1.03%		
No	572	98.62%	3045	98.83%			552	98.57%	2210	98.97%		
Cerebral infarction					3.82	0.042					0.8	0.224
Yes	18	3.10%	57	1.85%			17	3.04%	53	2.37%		
No	562	96.90%	3024	98.15%			543	96.96%	2180	97.63%		
Type of surgery					0.01	0.482					0.46	0.273
Emergency surgery	86	14.83%	2629	85.33%			80	14.29%	292	13.08%		
Elective surgery	452	77.93%	452	14.67%			480	85.71%	1938	86.79%		
Methods of anesthesia					8.61	0.035					0.55	0.908
General anesthesia	432	74.48%	2145	69.62%			418	74.64%	1674	74.97%		
Spinal anesthesia	77	13.28%	553	17.95%			76	13.57%	283	12.67%		
Nerve block	29	5.00%	178	5.78%			28	5.00%	110	4.93%		
Local anesthesia	42	7.24%	205	6.65%			38	6.79%	166	7.43%		
ASA Classification					26.95	<0.001					4.1	0.252
I	93	16.03%	721	23.40%			91	16.25%	426	19.08%		
II	438	75.52%	2194	71.21%			426	76.07%	1665	74.56%		
III	39	6.72%	145	4.71%			36	6.43%	126	5.64%		
IV	9	1.55%	21	0.68%			7	1.25%	16	0.72%		
V	1	0.17%	0	0.00%			0	0.00%	0	0.00%		

ASA: American society of Aneshesiologists; CHD: coronary heart disease; COPD: chronic obstructive pulmonary disease; DB: diabetes; DVT: deep vein thrombosis; HBP: hypertension; PSM: propensity score matching. *P* < 0.05 was considered statistically significant.

**Table 2:** Summary of patient characteristics after PSM

	Variance ± standard deviation	*P*-Value
Age (years)		0.423
Uninfected group	48.96±16.45	
0-7 days	50.91±16.2	
8-14 days	50.92±10.32	
14-30days	48.63±16.86	
>30days	46.89±17.39	
Medical expenses (yuan)		0.027
Uninfected group	30859.24±857.5	
0-7 days	39054.13±3386.6	
8-14 days	31753.43±6891.7	
14-30days	29852.9±2281.1	
>30days	29852.9 ±2442.6	

PSM: propensity score matching. *P* < 0.05 was considered statistically significant.

**Table 3:** Postoperative outcomes between SARS-CoV-2 negative patients and preoperatively positive SARS-CoV-2 patients stratified by timing of positivity before surgery with complication compared to SARS-CoV-2 negative patients

Variables	Uninfected group	Infected Group (560)					
	(2233) (REF)	0-7 days (317)	8-14 days (12)	15-30 days (117)	>30 days (130)	*X^2^*	*P*
Pulmonary infection/pneumonia	83(3.72%)	22(6.94%)	0	7(5.98%)	9(6.92%)	10.47	0.033
Pleural effusion	22(0.99%)	3(0.95%)	0	1(0.85%)	2(1.54%)	0.54	0.97
Postoperative DVT	30(1.34%)	6(1.89%)	0	2(1.71%)	2(1.54%)	0.82	0.935
Urinary tract infection	38(1.7%)	5(1.58%)	0	3(2.56%)	6(4.62%)	6.34	0.175
Low albumin	88(3.94%)	19(5.99%)	0	3(2.57%)	11(8.46%)	9.59	0.048
Atelectasis of lung	5(0.22%)	1(0.32%)	0	0	0	0.72	0.949
Anemia	164(7.34%)	23(7.26%)	0	5(4.27%)	18(13.85%)	10.31	0.035
Respiratory failure	14(0.63%)	9(2.84%)	0	0	1(0.77%)	17.07	0.002
Renal insufficiency	24(1.07%)	8(2.52%)	1(8.33%)	1(0.85%)	1(0.78%)	9.91	0.045
Sepsis	9(0.40%)	4(1.26%)	0	0	0	5.72	0.22
Blood transfusion	113(5.06%)	28(8.83%)	2(16.67%)	4(3.42%)	8(6.15%)	11.32	0.023
Transfer to ICU	43(2.84%)	9(2.84%)	0	2(1.71%)	2(1.54%)	1.61	0.807

DVT: deep vein thrombosis; ICU: Intensive care unit; PSM: propensity score matching. *P* < 0.05 was considered statistically significant.

## Abbreviations

ACE: Angiotensin converting enzyme
ASA: American society of Aneshesiologists
CHD: coronary heart disease
COPD: chronic obstructive pulmonary disease
DB: diabetes
DVT: deep vein thrombosis
HBP: hypertension
ICU: Intensive care unit
OR: Odd ratio
PCR: Polymerase chain reaction
PSM: propensity score matching
SARS-CoV-2: severe acute respiratory syndrome coronavirus 2
